# Insight into Molecular and Functional Properties of NMNAT3 Reveals New Hints of NAD Homeostasis within Human Mitochondria

**DOI:** 10.1371/journal.pone.0076938

**Published:** 2013-10-14

**Authors:** Roberta Felici, Andrea Lapucci, Matteo Ramazzotti, Alberto Chiarugi

**Affiliations:** Department of Health Sciences, University of Florence, Florence, Italy; Queen Mary University of London, United Kingdom

## Abstract

Among the enzymes involved in NAD homeostasis, nicotinamide mononucleotide adenylyltransferases (NMNAT1-3) are central to intracellular NAD formation. Although NMNAT3 is postulated to be a mitochondrial enzyme contributing to NAD-dependent organelle functioning, information on endogenous proteins is lacking. We report that in human cells a single gene nmnat3 localized on chromosome 3 codes for two mRNA splice variants NMNATv1 and FKSG76, whereas the previously reported NMNAT3v2 transcript is not present. However, NMNAT3v1 and FKSG76 proteins are not detectable, consistent with the finding that an upstream ORF in their mRNAs negatively regulates translation. NMNAT3v1 transfection demonstrates that the protein is cytosolic and inactive, whereas FKSG76 is mitochondrial but operates NAD cleavage rather than synthesis. In keeping with the lack of NMNAT3, we show that extracellular NAD, but not its metabolic precursors, sustains mitochondrial NAD pool in an ATP-independent manner. Data of the present study modify the scenario of the origin of mitochondrial NAD by showing that, in human cells, NMNAT3 is absent in mitochondria, and, akin to plants and yeast, cytosolic NAD maintains the mitochondrial NAD pool.

## Introduction

During the last several years, we witnessed a renewed interest in the biosynthesis of nicotinamide adenine dinucleotide (NAD) [1], [2]. This is due to the understanding that NAD homeostasis is not exclusively based on redox reactions (i.e. mutual conversion of NAD into NADH with conservation of the dinucleotide moiety) but also on the irreversible transformation of NAD into an expanding array of metabolites endowed with pleiotypic signaling properties [1], [3]. In keeping with this, several NAD-hydrolyzing enzymes such as poly (ADP-ribose) polymerases (PARPs), mono (ADP-ribose) transferases, sirtuins and the NADase CD38 have been identified. A great deal of investigation is now dedicated to the understanding of the physiological and pathological roles of these enzymes, with particular attention to development of pharmacological tools able to modulate their activities for therapeutic purposes [3]–[6].

A new concept of NAD homeostasis is that a major mechanism of regulation of NAD-consuming enzymes is availability of NAD itself within the different cell compartments [7], [8]. The understanding that, at variance with what previously envisaged, also metabolic, NAD-dependent redox reactions are limited by intracellular NAD concentrations significantly widens the pathophysiological and therapeutic implications of modulation of intracellular NAD availability [3]. In mammalian cells, the main pathway of NAD resynthesis is a two-step metabolic route. It stems from the conversion of nicotinamide (Nam) into nicotinamide mononucleotide (NMN) by nicotinamide phosphoribosyl-transferase, that is followed by the conversion of NMN into NAD by nicotinamide mononucleotide adenylyltransferase (NMNAT) [5]. At present, three NMNAT isoforms (NMNAT1-3) have been identified with different kinetic parameters and cellular localization [9]. While NMNAT1 is a nuclear enzyme responsible for the majority of NAD resynthesis [10], and NMNAT2 is a protein probably docked to the external surface of the endoplasmic reticulum contributing to cytosolic NAD rescue [11], less is known about NMNAT3.

NMNAT3 was initially identified by sequence homology with NMNAT1, cloned and localized in mitochondria, in keeping with the presence of a mitochondrial targeting sequence (MTS) [9], [12]. Catalytic mechanisms of NMNAT3 have been postulated [12], [13] but its functional relevance to NAD homeostasis and cell biology is almost unknown. This is because information on structural and catalytic properties of NMNAT3 has been exclusively collected by means of transient transfection in cultured cells [9], [12], [14]. Di Stefano et al. report the endogenous presence of NMNAT3 in red cells [15] that, however, do not contain mitochondria. On this basis, the question as to whether NMNAT3 is expressed in mammalian cells is still open. This is in striking contrast with the key role of mitochondrial NAD both to energetic metabolism and more integrated pathophysiological processes such as aging, obesity and diabetes [16].

In this light, we attempted to elucidate the functional relevance of endogenous NMNAT3 to mitochondrial NAD synthesis, with the aim of adding new information on how NAD homeostasis is maintained within the organelle.

## Materials and Methods

### Cell culture conditions and heat shock

HEK293 cells were cultured in Dulbecco's modified Eagle's medium (DMEM) supplemented with 2 mM glutamine, 10% fetal bovine serum and antibiotics. Cultures were brought to 50–70% confluence and used for the experiments. Cell cultures were exposed to NAD, Nam, NMN, nicotinamide riboside (NR), nicotinic acid (NA) or other compounds directly dissolved in the culture media. NR was synthesized as described by Yang et al. [17]. For heat shock experiments, cells were incubated 30 minutes at 44°C, and then subjected to different periods of recovery at 37°C. Later on, RNA was isolated and retro-transcribed as described below.

### NAD and ATP measurement

NAD contents were quantified by means of an enzymatic cycling procedure according to Cipriani et al. [18]. Briefly, cells grown in a 48 well plate were killed with 50 µl HClO_4_1N and then neutralized with an equal volume of KOH 1N. After the addition of 100 µl of bicine 100 mM pH 8, 50 µl of the cell extract was mixed with an equal volume of the bicine buffer containing 23 µl/ml ethanol, 0.17 mg/ml MTT, 0.57 mg/ml phenazine ethosulfate and 10 µg alcohol dehydrogenase. Mixture was kept at room temperature for 20 minutes and then absorbance a 550 nm was measured. A standard curve allowed quantification of NAD. The cellular ATP content was measured by means of an ATPlite kit from PerkinElmer Life and Analytical Sciences (Zaventem, Belgium).

### Cell fractionation and NMNAT activity assay

Mitochondria and nuclei were isolated from cells using a glass/glass homogenizer in 500 µl of extraction buffer, as described [19]. Briefly, supernatants were first centrifuged at 600 g to obtain nuclear fraction, and then were centrifuged at 7000 g to obtain the mitochondrial pellet. For enzymatic assay, cells, nuclear or mitochondrial fractions were disrupted with 200 µl of lysis buffer (25 mM Tris/HCl pH 8, 150 mM NaCl, 0.5% Triton-X). Extracts were incubated for 30 minutes at 37°C in a reaction mixture containing 50 mM Tris/HCl pH 8, 200 mM NaCl, 1 mM MgCl_2_ with 1 mM NMN and/or 1 mM ATP. NAD formation was evaluated using the above-mentioned enzymatic cycling procedure.

### Oxygen Consumption Analysis

Quantitation of oxygen consumption was conducted by means of the Oxygraph system (Hansatech Instruments, Norfolk, UK). Cells (250,000) were loaded in the chamber in 400 µl of respiration buffer (70 mM sucrose, 220 mM mannitol, 2 mM HEPES, pH 7.4, 5 mM MgCl2, 5 mM K2HPO4, 1 mM EDTA, and 0.1% bovine serum albumin), and oxygen consumption was monitored for 10 min at 37°C.

### Western and dot blotting

For Western blotting, cells were scraped, collected in Eppendorf tubes, centrifuged (1500 g/5 min/4°C) and resuspended in lysis buffer [50 mM Tris pH 7.4, 1 mM EDTA, 1 mM phenylmethylsulfonyl fluoride (PMSF), 4 µg/ml aprotinin and leupeptin, 1% SDS]. 20–40 µg of protein/lane were loaded. After 4–20% SDS-PAGE and blotting, membranes (Immobilon-P Millipore, Bedford, MA) were blocked with phosphate buffered saline (PBS) containing 0.1% Tween–20 and 5% skimmed milk (TPBS/5% milk) and then probed overnight with primary antibodies (1∶1000 in TPBS/5% milk). Dot blotting was conducted according to standard procedures with recombinant FKSG76 (kind gift of Prof. Mathias Ziegler, University of Bergen, Norway). Immunoprecipitation from mitochondrial or whole cell extracts has been conducted as reported [20] by means of a rat monoclonal antibody kindly gifted by Prof. Mathias Ziegler, University of Bergen, Norway. The anti-PAR monoclonal antibody (10H) and the anti-ubiquitin monoclonal antibody were from Alexis (Vinci, Italy). The anti NMNAT3 polyclonal antibody used for the experiments of Western and dot blotting was a kind gift of Prof. Giulio Magni (Università Politecnica delle Marche). Membranes were then washed with TPBS and incubated 1 h in TPBS/5% milk containing the corresponding peroxidase-conjugated secondary antibody (1∶2000). After washing in TPBS, ECL (Amersham, UK) was used to visualize the peroxidase-coated bands.

### PCR assays, cloning and transfection

Total RNA from HEK293 cells or human tissues was extract by means of RNeasy mini kit (Qiagen, Germany). Nucleic acid extracts digested with RNase-free DNase and resulting RNA quantified. One µg of RNA was retrotranscribed using iScript (Bio-Rad Milan Italy) amplified with specific primers described in Table 1. PCR products were separated on 2% agarose gel and the relative bands were analyzed by means of Sanger sequencing. Real-Time PCR was performed using Rotor-Gene 3000 (Qiagen Milan Italy) and the Rotor-Gene TM SYBR® Green PCR Kit (Qiagen, Milan, Germany). The following primers were used: for NMNAT1 forward 5′-TCCCATCACCAACATGCACC-3′ and reverse 5′-TGATGACCCGGTGATAGGCAG-3′; NMNAT3v1/FKSG76 forward 5′-ATGGGAAGAAAGACCTCGCAG-3′ and reverse 5′-AGTTTGCTGTGATGATGCCTC-3′;18S, forward 5′-CGGCTACCACATCCAAGGAA-3′ and reverse 5′-GCTGGAATTACCGCGGCT-3′. Primers were purchased from IDT (Leuven, Belgium). Cloning primers for NMNAT3v1 were: forward 5′- GGTACCGTTATGGGCTACCAGGTCATCCAGGGT-3′ and reverse 5′- GAACTCGAGCTAGCTTGTCTTGCCCTCAGTGC-3′. The amplification product from HEK293 cDNA was cloned into the pcDNA3.1+ vector (Invitrogen, Milan, Italy) containing the FLAG sequence. HEK 293 cells were transfected with 4 µg of pCDNA3-NMNAT3v1-FLAG, pFLAG5-FKSG76, mitoPARP1-cd or empty vectors. Silencing was obtained adopting a double-hit protocol exposing the cells to 50 nM siRNAs (Dharmacon, Lafayette, CO, USA) against NMNAT1 or NMNAT3at time 0 and 48 hrs. For cell transfection and silencing the jetPRIME™ kit (Polyplus, Illkirch, France) was used according to manufacturer's instructions.

**Table 1:** Primers adopted for RT-PCR experiments as depicted in Fig. 1A and C.

Primer	Sequence
1	5′ ATGAAGAGCCGAATACCTGTG 3′
2	5′ CTAGCTTGTCTTGCCCTCAG 3′
3	5′ CGAGACTCGCCGAGCGC 3′
4	5′ CTGGTACATTCCTTATTAATTGGGAGAC 3′
5	5′ CACCACAGGTACTCGGCTCTTC 3′

### Evaluation of upstream ORF functional activity

To amplify the 5′UTR sequence containing the upstream ORF (uORF) the following specific primers were used: forward AGAAGCTTTGCCATGAAGCGGACTGCTGCTC and reverse AACCATGGTATCAGGCACATCCACCC for FKSG76, or forward AGAAGCTTTGCCATGAAGCGGACTGCTGCTC and reverse AACCATGGTCCTTATTAATTGGGAGACAAG for NMNAT3v1. To mutagenize the uORF ATG we amplified with the forward primer AGAAGCTTTGCCGGGAAGCGGACTGCTGC containing GGG instead of the ATG start codon. PCR fragments were cloned into the HindIII and NcoI sites of the luciferase-expressing pGL3-Promoter Vector (Promega, Madison, WI) to obtain the following plasmids: pGL3-FKSG76-uORF, pGL3-FKSG76-Mut-uORF, pGL3-NMNAT3v1-uORF or pGL3-NMNAT3v1-Mut-uORF. HEK293 cells were co-transfected with pGL4,71 plasmid expressing renilla luciferase used as internal standard. Luciferase assay was performed as described by Lapucci et al. [21].

### Immunocytochemistry

Cells were grown onto glass coverslips and transfected with the different plasmids. After 48 hrs, cells were washed with PBS and then fixed with cold ethanol. After 1 h permeabilization and blocking in PBS 0.3% Triton X-100% containing 20 mg/ml BSA, cells were incubated for 2 h with PBS containing 5 mg/ml BSA plus the primary antibody diluted 1∶200. Anti-FLAG monoclonal antibody was from Sigma (Milan, Italy) and anti-PAR monoclonal antibody (10H) was from Alexis (Vinci, Italy). After extensive washing, cells were incubated for 45 min with the corresponding secondary antibody (1∶200 in PBS containing 5 mg/ml BSA) and washed again with PBS and mounted. Imaging was performed using a Nikon TE2000-U equipped with a Hg fluorescence lamp, a Photometrics CF mono CCD camera and Metamorph imaging software.

### Evaluation of mitochondrial membrane potential

Mitochondrial membrane potential was evaluated by means of flow cytometry [18]. Cells transfected with the empty vector or FKSG76 plasmid were then incubated with TMRE 2.5 nM in complete DMEM, detached and analyzed at the indicated time points. Briefly, cells were washed with PBS, incubated with trypsin (50 µl/0.25%/2 min) and then diluted with 350 µl complete DMEM. After gentle pipetting, 200 µl of the cell suspension were further diluted with 400 µl of PBS and analyzed by the flow cytometer Coulter EPICS XL (Beckman Coulter, Inc) equipped with the EXPO32 Flow Cytometry ADC software (Beckman Coulter, Inc). TMRE 2.5 nM was present in all the solutions used for cell preparation and measurement.

### Structural analysis of nmnat3 gene products

The PDB structure 1NUS coded by the ORF FKSG76 (UniProt code Q96T66) was analyzed. The 3D structure represents a 252 residue chain complexed with the ATP analog APC and NMN [10]. Structural investigations were drawn with Swiss PDB Viewer (DeepView) using hydrogen bond detection tools to evidence structural constraints in the full-length protein and mutation tools to create the structural variants described in this work and also provide valid templates for energetic minimization by the Swiss-Model 3D prediction server. Structures were also investigated using the fold recognition servers Robetta and Phyre.

### Detection of NAD content by mitoPARP-1cd

Mitochondrial NAD content was evaluated using the mitoPARP-1cd construct as described [22]. Briefly, cells were transfected with mitoPARP-1cd and/or FKSG76. After 48 hrs, PAR formation was evaluated by means of Western blot analysis. Variations in the extent of detected PAR reflect changes of the mitochondrial NAD content. Treatment with NAD or its precursors began 48 hrs prior to PAR analysis.

### Data analysis

Data were analysed using WinLTP 1.11 reanalysis program and the software package GraphPad Prism (version 4.0; GraphPad Software, San Diego, CA, USA). All numerical data are expressed as mean ± SEM. Statistical significance was evaluated using paired two-tailed Student's t-test. Differences were considered significant at *p*<0.05.

## Results

### Identification of NMNAT3 variants expressed in human cells

**Figure 1:**
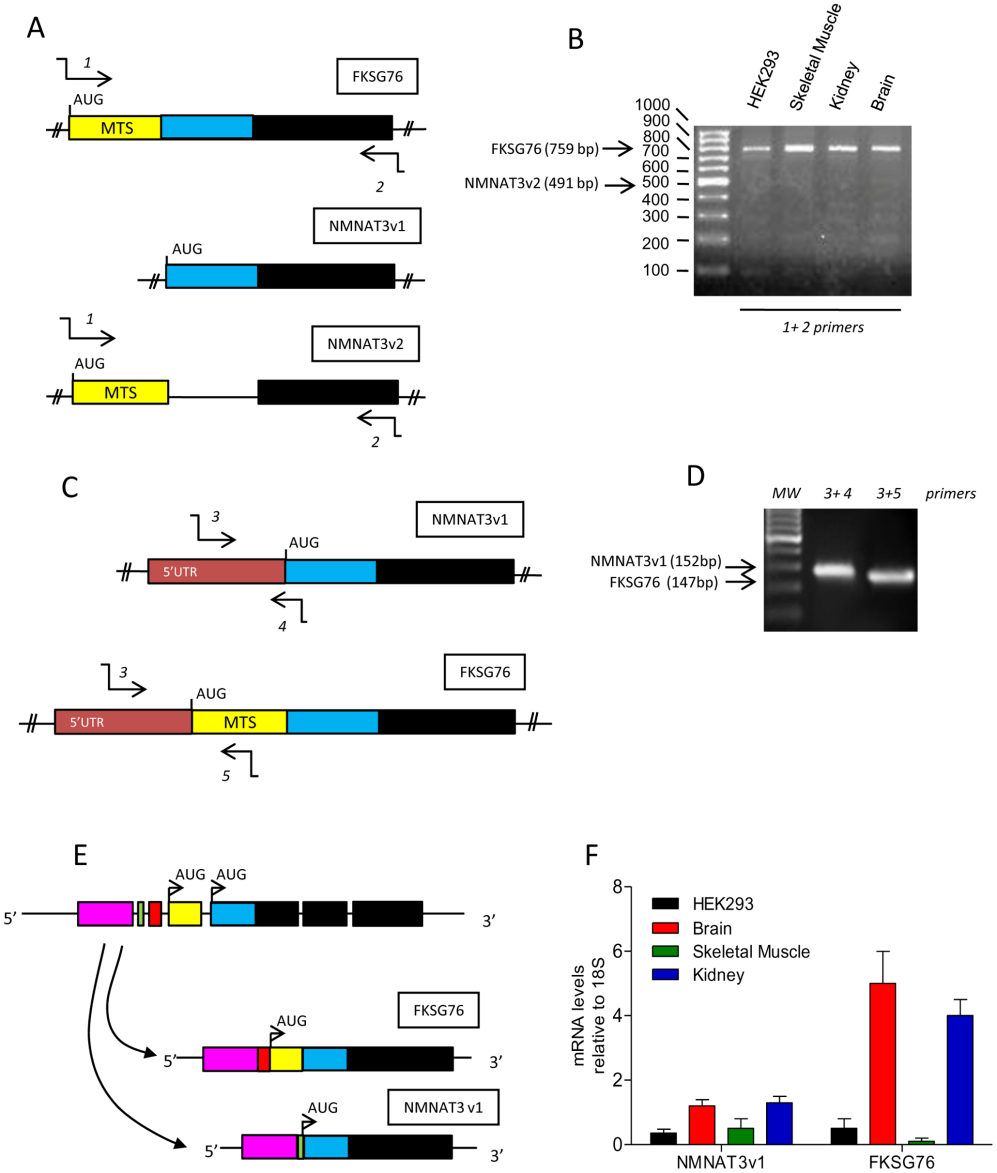
Expression of NMNAT3 mRNA variants in human cells. (A) Schematic representation of the ORF of FKSG76, NMNAT3v1 and NMNAT3v2. Colors represent different homology domains. MTS, mitochondrial targeting sequence. Annealing position of primers 1 and 2 (Table 1) used to amplify the ORF of FKSG76 and NMNAT3v2 is shown. (B) Semiquantitative PCR showing the presence of the band of 759 bp related to FKSG76 ORF and the absence of that of 491 bp related to amplification of NMNAT3v2 ORF in HEK293 cells and human brain, skeletal muscle and kidney tissues. (C) Schematic representation of a portion of NMNAT3v1 and FKSG76 transcripts containing their 5′UTR. Colors represent homology domains. Annealing position of primers 3, 4 and 5 (Table 1) used to amplify the fragments of FKSG76 and NMNAT3v2 is shown. (D) Semiquantitative PCR showing the presence of the expected bands of 152 and 147 bp related to amplification of the regions of NMNAT3v1 and FKSG76 shown in (C). (E) Schematic reconstruction of the pre-mRNA structure from which FKSG76 and NMNAT3v1 transcripts originate by alternative splicing. Colors represent homology domains. (F) Comparative analysis of FKSG76 and NMNAT3v1 transcript levels in different human tissues. Agarose gels are representative of at least 4 independent experiments. In (F) columns represent the mean ± SEM of 3 RT-PCR experiments with different human samples.

In an attempt to understand the structure of the gene coding for NMNAT3, we came across to apparent inconsistencies present in GenBank. According to Zhang and associates, NMNAT3 is encoded by the transcript FKSG76 originating from the gene fksg76 located on chromosome 8 (accession AF345564) [12]. However, an NCBI search localizes nmnat3 on chromosome 3 coding for two different transcript variants called NMNAT3v1 (accession NM_178177.3) and NMNAT3v2 (accession NM_001200047.1). We therefore attempted to confirm the presence of fksg76 on chromosome 8 by means of NCBI nucleotide blast search, and found that the mRNA exclusively matches with a sequence present on chromosome 3. We then compared FKSG76, NMNAT3v1 and NMNAT3v2 mRNAs and found substantial sequence homology (see Fig. 1A for a schematic representation). Of note, a nucleotide sequence coding for the mitochondrial targeting sequence (MTS) is present in FKSG76 and NMNAT3v2 mRNAs but absent in that of NMNAT3v1, thereby suggesting a different intracellular localization of the latter. To gather additional information on NMNAT3 variant expression, we attempted to amplify mRNAs of FKSG76, NMNAT3v1 and NMNAT3v2 from cDNA of HEK293 cells by means of RT-PCR and specific primers (Table 1). We first used primers putatively amplifying the entire ORF of FKSG76 and NMNAT3v2 (that would have different molecular weights, see Fig. 1A), and found that only the amplification product with the expected molecular weight of FKSG76 was obtained, but not NMNAT3v2 (Fig. 1B). NMNAT3v2 transcripts were also absent in human tissues such as brain, skeletal muscle and kidney (Fig. 1B). Next, to check for the presence of NMNAT3v1 mRNA, we designed a forward primer annealing within its 5′UTR, and a reverse one annealing on a region encompassing the start codon (Fig 1C). We found an amplification product of the expected molecular weight (Fig. 1D), that, upon Sanger analysis, confirmed the sequence of NMNAT3v1 present in GenBank. In light of the apparent mislocalization of fksg76 on chromosome 8 (see above), and the high degree of sequence homology between NMNAT3v1 and FKSG76 (Fig. 1A), we then speculated that these transcripts could be splice variants. Consistent with this hypothesis, when we used the above-mentioned forward primer binding to the 5′UTR of NMNAT3v1 and a reverse one binding inside the MTS of FKSG76 (Fig. 1C), a fragment of 147 bp was obtained (Fig. 1D). The amplification product showed the expected 5′UTR sequence identical to that of NMNAT3v1 and the MTS sequence of FKSG76 plus a fragment of 39 bp comprised between these two regions (Figure S1). These data taken together indicate that nmnat3 on chromosome 3 codes for a pre-mRNA from which FKSG76 and NMNAT3v1 but not NMNAT3v2 transcripts originate by alternative splicing (Fig. 1E).

Real time PCR showed that NMNAT3v1 and FKSG76 mRNAs were differently transcribed in HEK293 cells and human tissues, with FKSG76 transcripts showing high levels in brain and kidney (Fig. 1F).

### Effect of NMNAT1 and −3 silencing on cellular NMNAT activity

**Figure 2:**
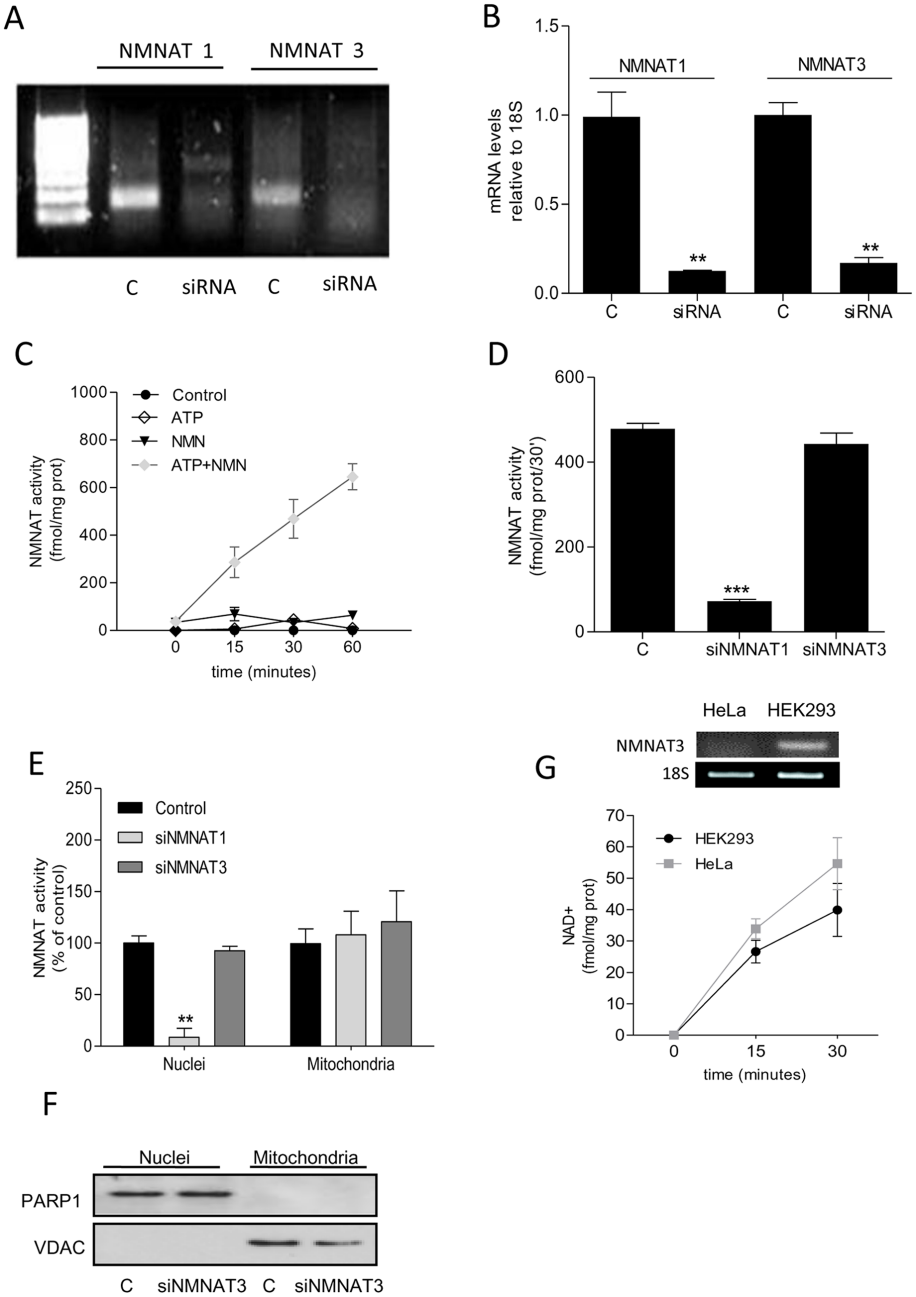
Effect of NMNAT1 or −3 silencing on cellular NMNAT activity. (A) Semiquantitative PCR showing silencing of NMNAT1 or NMNAT3 by their respective siRNAs. Note that for NMNAT3 siRNAs able to anneal a sequence present in both NMNAT3v1 and FKSG76 have been used. For NMNAT3 PCR, a sequence present in both NMNAT3v1 and FKSG76 has been amplified. (B) Real-time PCR analysis of the effect of NMNAT3v1 or FKSG76 silencing. (C) NMNAT activity in whole HEK cell extracts and different combinations of the substrates. (D) Effect of NMNAT1 or NMNAT3 silencing on cellular NMNAT activity. (E) Effect of NMNAT1 or NMNAT3 silencing on nuclear or mitochondrial NMNAT activity. Nuclear and mitochondrial NMNAT activities were 2430±390 and 37±7 Fmol/mg prot/30′, respectively. (F) Western blotting evaluation of PARP-1 and VDAC in the nuclear and mitochondrial fractions of control and silenced HEK293 cells. (G) NMNAT3 transcript levels and mitochondrial NMNAT activity in HeLa and HEK cells. In (A) and (G) an experiment representative of 5 is shown. Columns/points represent the mean ± SEM of at least 4 experiments. ** p<0.01; *** p<0.001 vs control (Student's t test).

As mentioned above, functional relevance of endogenous NMNAT3 to cellular and mitochondrial NAD homeostasis is currently unknown. Thus, to gather insights into the contribution of NMNAT3 to NAD homeostasis, we silenced NMNAT3 by means of siRNA designed to reduce transcripts of both FKSG76 and NMNAT3v1. We also evaluated the effects of NMNAT1 silencing. Fig 2A and B show that siRNAs for NMNAT1 or NMNAT3 drastically reduced transcript levels after 72 hrs. We therefore adopted an enzymatic assay measuring NAD formation from NMN and ATP to check whether silencing of NMNAT1 or −3 reduced cellular NMNAT activity. The assay was specific for NMNAT activity being able to detect NAD formation by a whole cellular homogenate only in the presence of added ATP and NMN (Fig. 2C). Of note, we used HEK293 cells because they express negligible transcripts for NMNAT2 [9], thereby allowing to ascribe their NMNAT activity to NMNAT1 and −3 only. We found that cellular NMNAT activity was significantly reduced in cells subjected to NMNAT1 but not NMNAT3 silencing (Fig. 2D). In keeping with the nuclear localization of NMNAT1, subcellular evaluation of NMNAT activity showed that only the nuclear fraction was depleted of enzymatic activity in cells challenged with siRNA for NMNAT1. Conversely, no differences in NMNAT activity were found in nuclear or mitochondrial fractions of cells exposed to NMNAT3 silencing (Fig. 2E). As evidence of purity of the subcellular fractions, Western blotting demonstrated exclusive localization of PARP-1 and VDAC in the nuclear and mitochondrial extracts, respectively (Fig. 2F). Data suggest that NMNAT3 activity does not contribute to mitochondrial NMNAT activity. According to this assumption, we found that HeLa cells showed levels of mitochondrial NMNAT activity analogous to those of HEK293 cells, despite lack of NMNAT3 transcripts (Fig. 2G).

### Effect of overexpression of NMNAT3 isoforms on cellular NMNAT activity and NAD homeostasis

**Figure 3:**
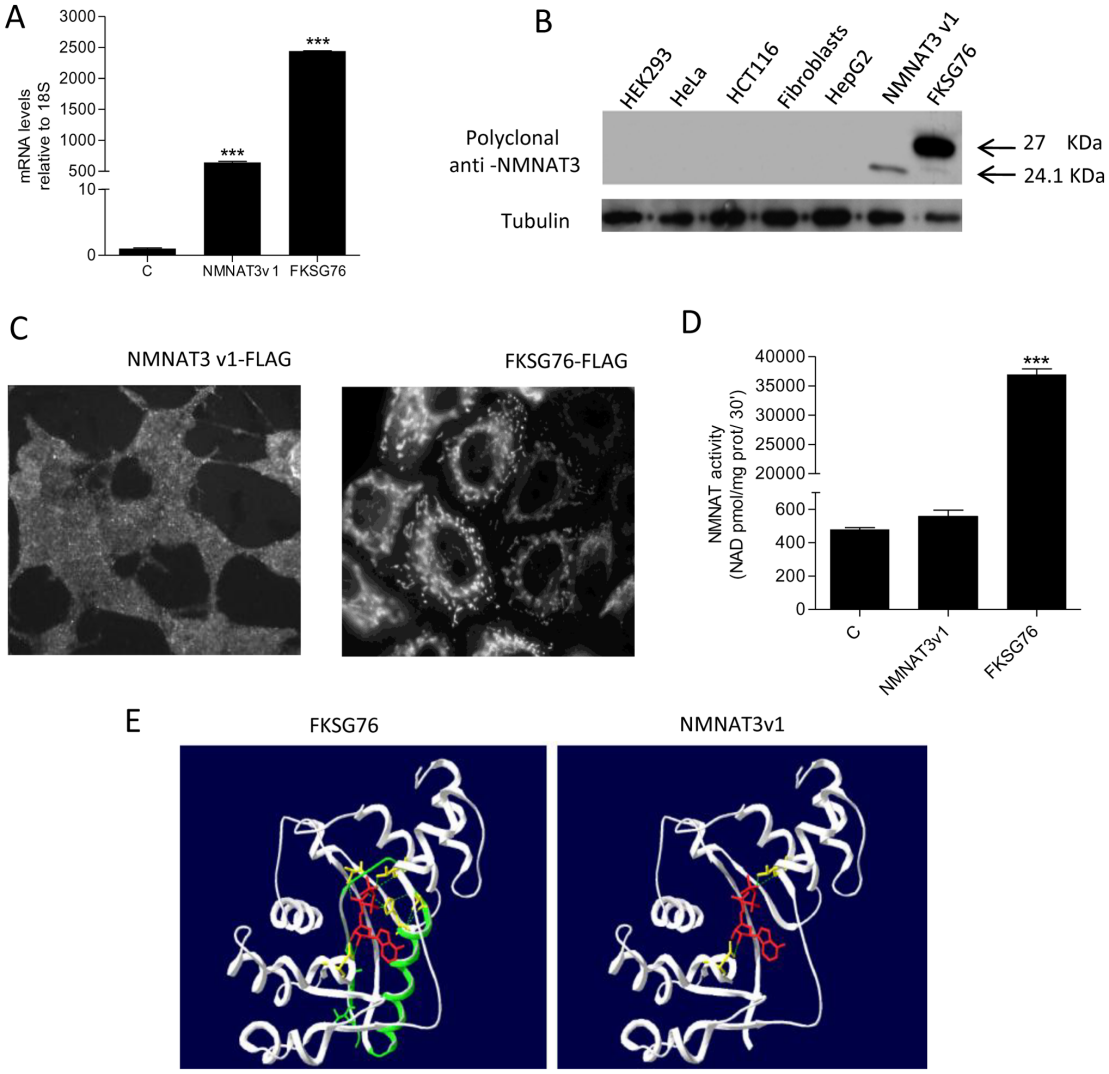
Expression, intracellular localization and structure of transfected FKSG76 and NMNAT3v1. (A) Fold increase of mRNA for NMNAT3v1 or FKSG76 upon transfection of the respective expression plasmids. (B) Western blotting analysis of NMNAT3v1 or FKSG76 expression in different cell types as well as NMNAT3v1- and FKSG76-transfected HEK cells. Tubulin is shown as loading control. (C) Immunocytochemical visualization of intracellular distribution of transfected FKSG76 or NMNAT3v1 by means of anti-FLAG antibody. (D) Whole cell NMNAT activity in NMNAT3v1 or FKSG76 transfected cells. (E) Structure of FKSG76 and NMNAT3v1. The ATP-binding domain absent in NMNAT3v1 is shown in green in FKSG76. ATP-binding residues are shown in yellow. The orientation of ATP (red) bound into the catalytic site is also shown. Columns represent the mean ± SEM of 3 (A) and 4 (D) experiments. Western blotting and Immunocytochemistry are representative of 4 independent experiments. *** p<0.001 vs control (Student's t test).

To obtain further insights on the effect of the two NMNAT3 isoforms on NAD homeostasis, we next investigated the impact of FKSG76 or NMNAT3v1 transfection on various cellular parameters. Transcripts for FKSG76 or NMNAT3v1 increased about 600- and 2400-fold in transfected cells (Fig. 3A). By means of a polyclonal antibody raised against recombinant NMNAT3 [15], we then checked for expression of the two NMNAT3 isoforms in control or transfected HEK cells. By means of Western blotting, we found that protein expression of both FKSG76 and NMNAT3v1 did not reach detection limit in control cells. Similar results were obtained in extracts from human cell lines such as HeLa (cervix), HCT116 (colon), fibroblasts and HepG2 (liver) (Fig. 3B). Conversely, bands of the expected molecular weight of 27 and 24 kDa appeared in extracts from cell transfected with FKSG76 and NMNAT3v1, respectively (Fig. 3B). Intracellular distribution of the two tagged NMNAT3 variants was also evaluated by anti-FLAG immunocytochemistry. In keeping with the exclusive presence of the MTS sequence in FKSG76 (Fig. 1C), NMNAT3v1 was evenly distributed throughout the cell, conversely, as previously reported [9], FKSG76 showed a mitochondrial localization (Fig. 3C).

**Figure 4:**
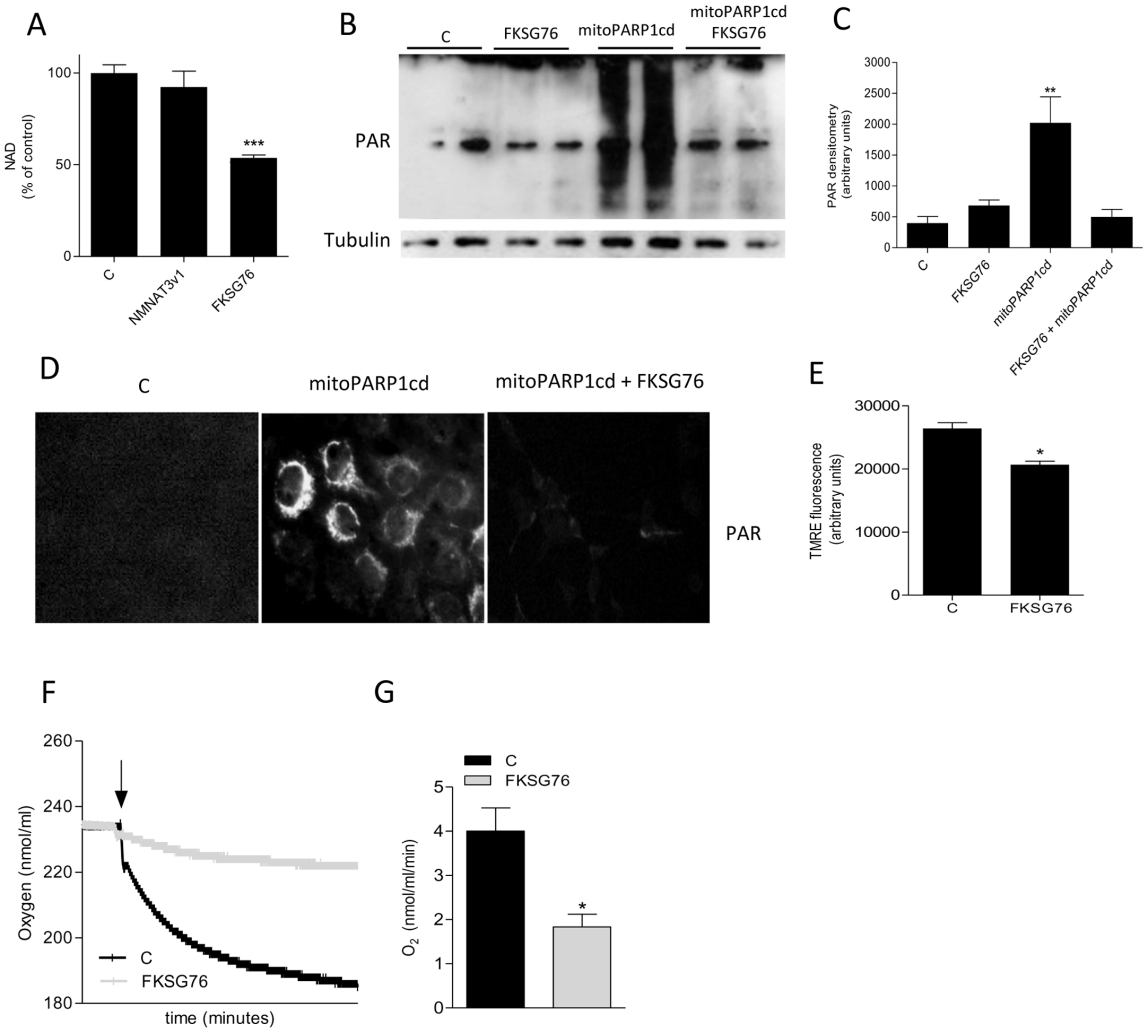
Effect of NMNAT3v1 or FKSG76 transfection on cellular or mitochondrial NAD and membrane potential. (A) Whole cellular NAD content in control, NMNAT3v1- and FKSG76-transfected cells. Basal NAD content was 12.6±2 nmol/mg prot. (B) Western blotting evaluation of poly(ADP-ribose) (PAR) formation in HEK cells under control conditions or after transfection of FKSG76 and/or PARP1-cd. Tubulin is shown as loading control. (C) Densitometric evaluation of PAR formation shown in (B). (D) Immunocytochemical localization of PAR in HEK cells under control conditions or after transfection of FKSG76 and/or mitoPARP1cd. (E) Effect of FKSG76 transfection on mitochondrial membrane potential. (F) Representative experiment of the effect of FKSG76 transfection on oxygen consumption. The arrow indicates the time when cells were added to the respiration buffer. (G) Oxygen consumption rate in control or FKSG76-transfected cells. Columns represent the mean ± SEM of 4 (A), 5 (C) and 3 (G) experiments. Western blotting and immunocytochemistry are representative of 5 and 2 experiments, respectively.* p<0.05; ** p<0.01; *** p<0.001 vs control (Student's t test).

We then investigated the contribution of NMNAT3v1 or FKSG76 overexpression to cellular NMNAT3 activity. NMNAT activity was not increased in cells transfected with NMNAT3v1, whereas an almost 800-fold increase was evident in those transfected with FKSG76 (Fig. 3D). A subsequent in silico analysis (see Material and Methods) of NMNAT3v1 3D structure revealed that the protein lacks the region required to bind and orient ATP for enzymatic catalysis (Fig. 3E), thereby explaining why NMNAT activity is not increased in cells transfected with NMNAT3v1. Consistently, NAD contents did not change in NMNAT3v1-transfected cells. Unexpectedly, however, FKSG76 overexpressing cells showed almost half of the basal cellular content of NAD (Fig. 4A).

To understand whether FKSG76 overexpression reduced mitochondrial NAD content, we took advantage of a recent method developed to indirectly quantify NAD levels within the organelle [22]. Specifically, we evaluated NAD-dependent poly (ADP-ribose) (PAR) content in cells transfected with a mitochondrially-targeted catalytic domain of PARP-1 (mitoPARP1cd) that leads to substantial PAR formation within mitochondria. By means of Western blotting, we found that PAR content drastically increased in mitoPARP1cd-transfected cells, and that this increase was completely abrogated in those co-transfected with FKSG76 (Fig. 4B and C). Immunocytochemistry confirmed prevention of mitochondrial PAR formation by FKSG76 (Fig. 4D). Given that mitochondrial NAD is a key determinant of organelle bioenergetics, we evaluated the effect of FKSG76 transfection on mitochondrial membrane potential and oxygen consumption. In keeping with reduced mitochondrial NAD content, cells overexpressing FKSG76 showed reduced mitochondrial membrane potential (Fig 4E) and oxygen consumption (Fig. 4F and G).

### Further attempts to identify endogenous NMNAT3 expression

**Figure 5:**
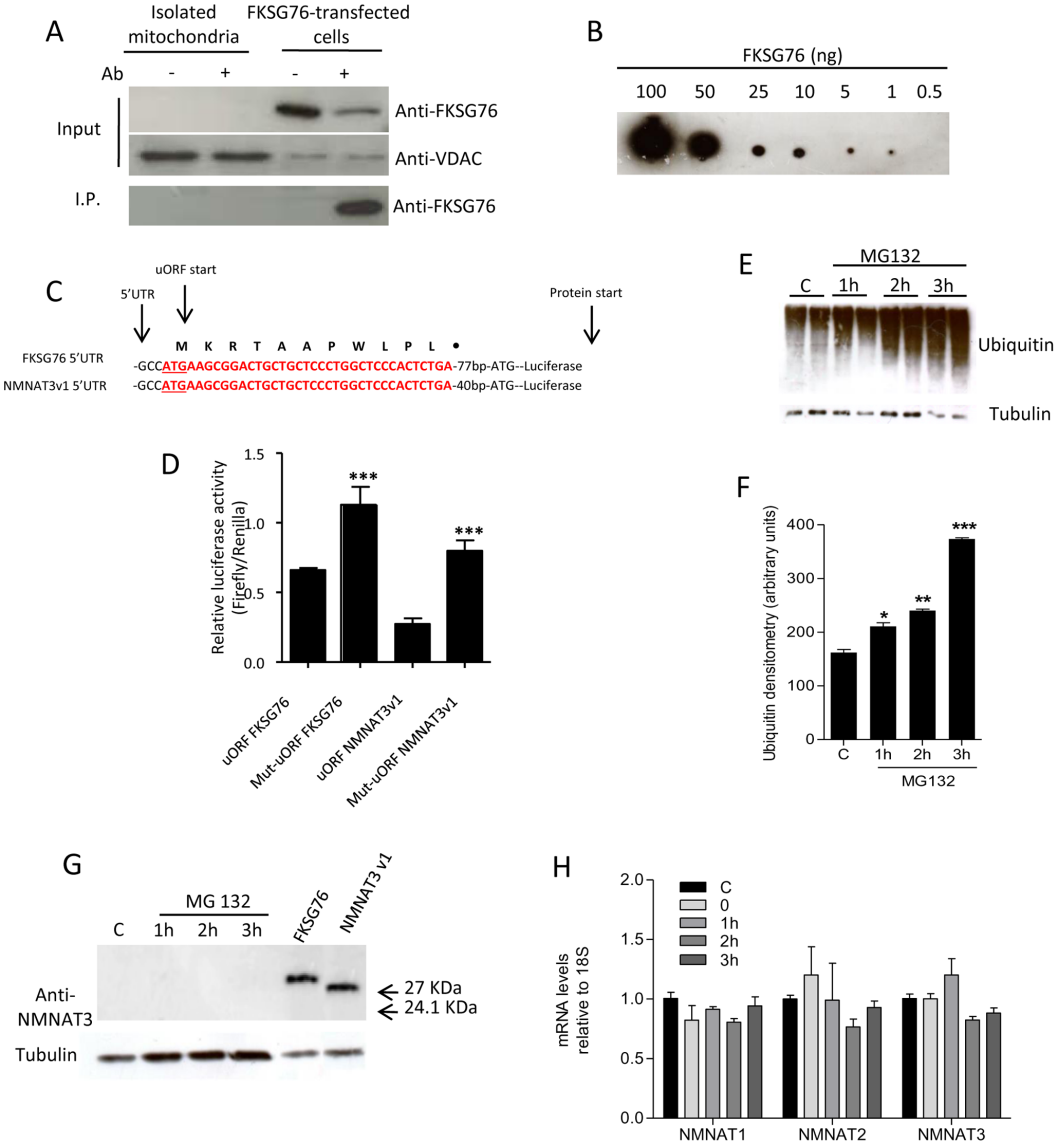
Identification of FKSG76 and NMNAT3v1 expression under different cellular conditions. (A) Immunoprecipitation of NMNAT3 from mitochondrial extracts of HEK239 cells or FKSG76-transfected whole cell homogenate. VDAC is shown as a mitochondrial marker. (B) Dot-blot evaluation of the sensitivity of anti-NMNAT3 antibody to recombinant FKSG76. (C) Schematic representation of the 5′UTRs of FKSG76 and NMNAT3v1 cloned in the reported vector used in Luciferase reporter assay (See Methods); the uORF sequences are depicted in red. (D) Effect of uORFs or mutated uORFs (Mut-uORFs) on translational efficiency. (E) Time-dependent accumulation of ubiquitinated-proteins in MG132-exposed (10 µM) cells. Tubulin is shown as loading control. (F) Densitometric evaluation of ubiquitin accumulation shown in (E). (G) Western blotting evaluation of FKSG76 and NMNAT3v1 expression in HEK cells after different times of exposure to the proteasome inhibitor MG132. Positive control for FKSG76 or NMNAT3v1 are shown. Tubulin is shown as loading control. (H) Transcript levels of NMNAT1, −2 and −3 at different times after heat shock (44°C/30′). Note that for NMNAT3 analysis primers able to amplify a common region of NMNAT3v1 and FKSG76 have been used. Columns represent the mean ± SEM of 3 experiments. Western blotting or Dot-blot are representative of at least 3 experiments.* p<0.05; ** p<0.01; *** p<0.001 vs control (Student's t test).

To rule out the possibility of excessive FKSG76 dilution in cell extracts, we attempted to immunoprecipitate the protein from mitochondrial extracts by means of a rat monoclonal antibody raised against FKSG76. As shown in Fig. 5A, FKSG76 was not detected neither in the input mitochondrial extract nor in the immunoprecipitate. Conversely, the protein was present in the immunoprecipitate from FKSG76-transfected cells. Dot blot analysis of the antibody used to detect FKSG76 and NMNAT3v1 revealed that threshold sensitivity for FKSG76 was in the range of 0.5 ng (Fig. 5B). In depth analysis of the 5′UTR regions of FKSG76 and NMNAT3v1 mRNAs revealed the presence of an upstream ORF (uORF) in both transcripts (Figure S1). uORFs impair translational efficiency of the downstream coding sequence because it can lead to ribosome stalling and mRNA decay [23], [24]. Thus, we studied the functional effects of the uORFs found in FKSG76 and NMNAT3v1. To this end, we cloned the 5′UTR fragments of FKSG76 and NMNAT3v1 bearing either the wild type uORF or a mutagenized one (converting the ATG into GGG) into luciferase expression vectors (see Fig. 5C). Remarkably, disruption of the wild type uORF significantly increased luciferase expression (Fig. 5D). These findings suggest that uORFs of FKSG76 and NMNAT3v1 transcripts impair their translation, in keeping with our inability to detect the respective proteins under control conditions.

Thus, in an attempt to boost FKSG76 and NMNAT3v1 expression, we exposed HEK cells to different stimuli. We first reasoned that their undetectable expression levels might be due to rapid proteasome-dependent degradation. Fig. 5E and 5F indicate that, upon exposure of HEK cells to the proteasome inhibitor MG132, intracellular ubiquitin levels linearly increased with time, indicating efficacy of the drug. Yet, we did not detect expression of FKSG76 or NMNAT3v1 in cells exposed to MG132 (Fig. 5G).

Recent findings demonstrate that *Drosophila* NMNAT behaves as a chaperone protein and can be induced by heat shock [25], [26]. To investigate whether human NMNATs are inducible proteins, we then studied the effect of heat shock on their transcripts. As shown in Fig 5H 44°C/30′ heat shock did not affect transcript levels for NMNAT3 (primers able to amplify both FKSG76 and NMNAT3v1 were used) or NMNAT1, and −2, thereby ruling out that NMNATs are inducible proteins in human cells.

### Effect of exogenous NAD and its precursors on cellular and mitochondrial NAD content in FKSG76-overexpressing cells

Although NMNAT3 is thought to contribute to mitochondrial NAD homeostasis, data of the present study challenge this scenario. Recent reports demonstrate that exogenous NAD increases the mitochondrial dinucleotide pool [8], [27]. We therefore checked whether the dinucleotide or its precursors added to the culture media were able to prevent NAD depletion in cells transfected with FKSG76.

**Figure 6:**
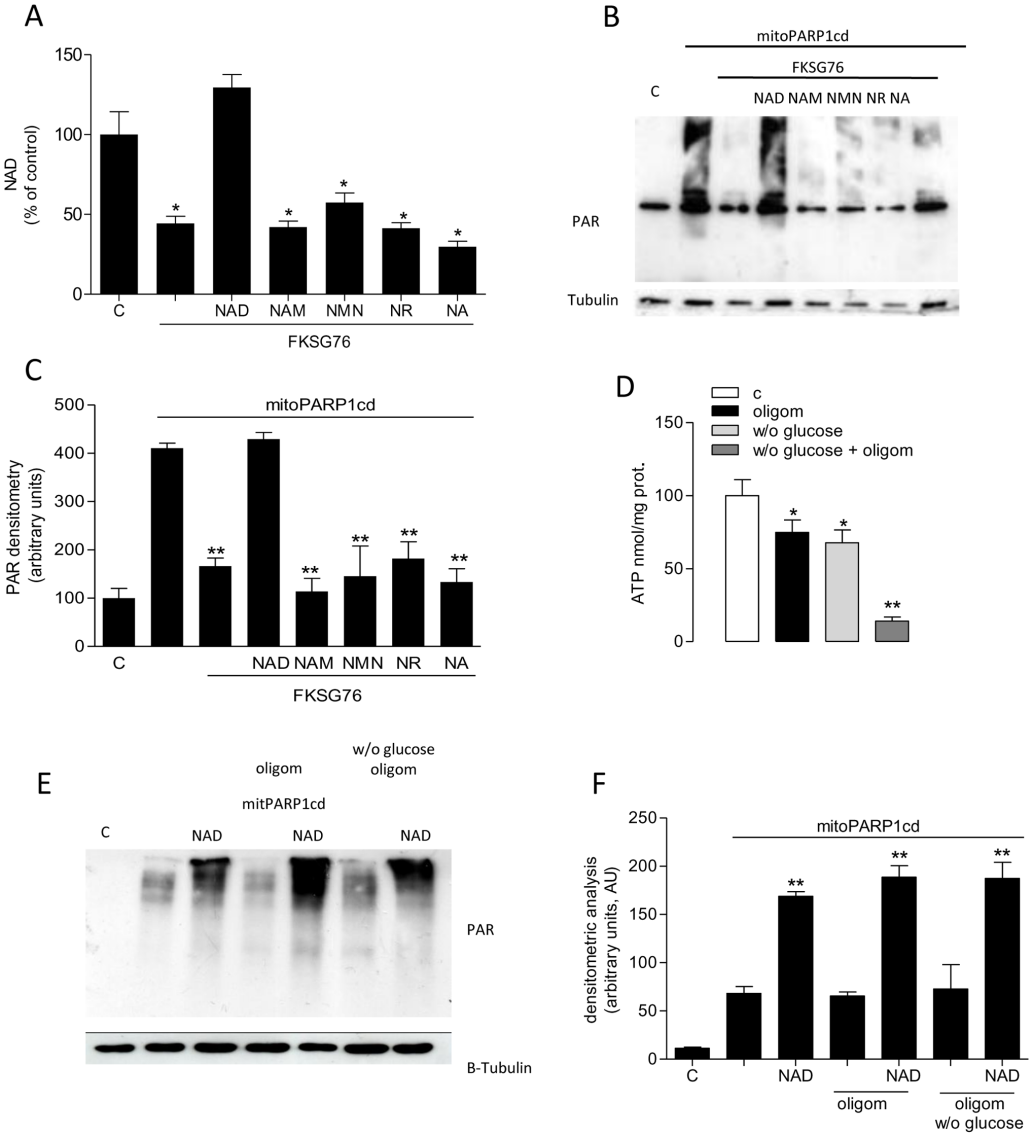
Effect of exogenous NAD or its precursors on cellular and mitochondrial NAD. (A) Quantification of NAD in control or FKSG67-transfected HEK cells. The effect of NAD, nicotinamide (Nam), NMN, nicotinamide riboside (NR) or nicotinic acid (NA) on NAD contents of FKSG-transfected cells is shown (NAD and its precursors have been added to the incubating media at 1 mM for 48 hrs). Basal NAD content was 12.6±2 nmol/mg prot. (B) Western blotting evaluation of the effect of NAD, Nam, NMN, NR or nicotinic acid NA (1 mM/48 hrs) on depletion of mitochondrial PAR content induced by FKSG76 co-transfection in mitoPARP1cd-transfected cells. Tubulin is shown as loading control. (C) Densitometric analysis on the experiment shown in (B). (D) Effect of oligomycin (10 µM/30 min) and/or glucose deprivation (30 min) on cellular ATP contents. (E) Effects of exogenous NAD (1 mM/3 hrs) on mitochondrial PAR contents in mitoPARP1cd-transfected cells under control conditions or exposed to oligomycin (10 µM) in the presence or absence of glucose. (F) Densitometric analysis on the experiment shown in (E). Columns represent the mean ± SEM of 3 experiments. Western blotting is representative of 3 (E) and 4 (C) experiments.* p<0.05; ** p<0.01 vs control (Student's t test).

Intriguingly, we found that cellular NAD depletion could be completely prevented by adding 1 mM NAD to the culture media (Fig. 6A). However, identical concentrations of the NAD precursors Nam, NMN, nicotinamide riboside and nicotinic acid were not effective (Fig. 6A). By means of the mitoPARP1cd assay, we also investigated whether exogenous NAD and its precursors prevented mitochondrial NAD depletion. Again, we found that exogenous NAD fully prevented reduction of PAR formation in mitochondria of cells co-transfected with FKSG76 and mitoPARP1cd, an effect not mimicked by NAD precursors (Fig. 6B, 6C). Evidence that the NAD effects were not reproduced by its precursors, suggested that NAD directly crosses the mitochondrial to maintain the organelle's NAD contents. To further corroborate this assumption, we then reasoned that if mitochondrial NAD formation occurs by matrix condensation of NMN with ATP by NMNAT activity, then mitochondrial ATP depletion should impair intraorganelle NAD biosynthesis. To verify this hypothesis, we blocked mitochondrial ATP formation with the F1Fo ATP synthase inhibitor oligomycin. The latter almost completely reduced ATP contents in cells cultured in the absence of glucose (Fig. 6D), in keeping with inhibition of mitochondrial ATP formation. Notably, under these conditions the ability of exogenous NAD to increase intramitochondrial PAR formation in mitoPARP1cd-transfected cells was unaffected (fig. 6E, 6F), suggesting that the NAD pool of mitochondria can be sustained by direct entrance of cytosolic NAD, and does not require ATP-dependent enzymatic steps.

## Discussion

The present study sought to investigate the functional relevance of NMNAT3 to mitochondrial NAD homeostasis. At variance with prior information present in GenBank, we now report that a single gene present on chromosome 3 codes for the two mRNA splice variants NMNAT3v1 and FKSG76. However, we have been unable to find molecular or functional evidence for endogenous expression of the respective proteins. When transfected, NMNAT3v1 appears cytosolic and inactive, whereas FKSG76 localizes into mitochondria and reduces, rather than increases, the organelle NAD content. Finally, we provide evidence that exogenous NAD, but not its metabolic precursors, is able to prevent mitochondrial NAD pool depletion caused by transfection of FKSG76.

NMNAT3 has been first identified by means of sequence homology with NMNAT1 [12]. Later on, almost all the information collected on NMNAT3 localization and activity has been obtained by expression plasmids coding for tagged proteins [9], [12], [14]. The group of Magni, however, reported the presence of NMNAT3 protein in human red blood cells. Intriguingly, as the authors point out, reticulocytes undergo complex events of mRNA maturation during differentiation that theoretically could underlie cell-specific processing of pre-mRNA of nmnat3 [15]. An additional study by Barile et al. reports the presence of NMNAT activity in the mitochondrial matrix of rat hepatocytes [28]. It is worth noting, however, that NADH rather than NAD was measured in this as product of NMNAT. Furthermore, Barile and associates report that exogenous NMN fuels mitochondrial NMNAT activity only in conditions of organelle permeabilization [28], indicating that the mononucleotide *per se* does not cross the inner mitochondrial membrane. Although caution must be exercised when interpreting these data as evidence for the presence of a *bona fide*, mitochondrial NMNAT, recent work suggests that cytosolic NMN is the precursor of mitochondrial NAD [27]. In keeping with the study by Barile et al. [28], we found a NAD-synthesizing activity from NMN and ATP in mitochondrial extracts (Fig. 2E, 2F). However, given that mitochondrial NMNAT activity is not affected by concomitant silencing of FKSG76 and NMNAT3v1 (Fig. 2E) and NMNAT activity is present in HeLa cells in spite of lack of NMNAT3 transcripts (Fig. 2G), an alternative interpretation should be put forward. In particular, mitochondria might contain an enzymatic activity, possibly belonging to the superfamily of nucleotidyltransferaseα/βphosphodiesterases [29], able to catalyze nucleotidylation of NMN but with a different physiological role. In keeping with this hypothesis, Km of mitochondrial NMNAT activity for NMN reported by Barile and coworkers [28] is 18,2 µM whereas that of recombinant NMNAT3 is 209 µM [9].

Data obtained by means of FKSG76 overexpression in control or mitoPARP1cd-transfected cells indicate that this protein is indeed targeted to mitochondria but operates in a reverse mode (i.e. NAD cleavage). Of note, this finding is in good agreement with prior work indicating that Vmax of FKSG76 for NAD cleavage is almost 4-fold higher than that for NAD synthesis (12.8 and 3.6 µmol/min/mg prot, respectively) [9]. Exact knowledge of concentrations of free ATP, NMN, NAD and PPi within the mitochondrial matrix is currently lacking. However, the poor permeability of mitochondrial membrane to NMN [28] may limit the availability of this NMNAT3 substrate within the organelle. This homeostatic conditions, together with substantial mitochondrial content of NAD and PPi, could well explain why FKSG76 operates in a reverse mode when artificially present in mitochondria. In light of the key role of NAD in mitochondrial respiration and overall organelle functioning, it is also conceivable that a protein able to deplete the mitochondrial NAD pool such as FKSG76 can severely jeopardize organelle homeostasis and cell survival. Accordingly, we found that endogenous FKSG76 cannot be detected by Western blotting or immunoprecipitation, suggesting that it is not translated. Dot blot analysis demonstrated that the antibody used to detect the two NMNAT3 isoforms has a threshold sensitivity in the range of 0.5 ng. This is consistent with sensitivity of antibodies normally used for Western blotting, and further suggests that FKSG76 and NMNAT3v1 are not expressed. Of course, we cannot rule out the possibility that these two proteins are expressed at a very low level, that, however, is not of functional significance (as the silencing experiments indicate). Still, even a very recent, elegant proteomic approach able to identify the whole mitochondrial proteome does not report NMNAT3 among the organelle proteins [30]. In keeping with lack of FKSG76 and NMNAT3v1 protein expression, in the 5′UTR of their mRNAs we identified a common uORF that reduces translational efficiency of the downstream ORFs. The quantitative impact of this uORF on endogenous FKSG76 and NMNAT3v1 translation is unknown. Still, considering that it is able to significantly impair luciferase expression driven by the very potent SV40 promoter, we reason that its negative impact on physiological FKSG76 and NMNAT3v1 expression should be of functional significance. Accordingly, complete uORF-dependent suppression of protein expression is reported [24]. Interestingly, the high degree of conservation of uORF start codons in humans is considered as evidence of key, albeit unknown, functional roles [23], [24]. We speculate that a possible reason for presence of uORF in transcripts of NMNAT3 variants is that of preventing FKSG76-expression within mitochondria and ensuing deleterious NAD depletion. Alternatively, these transcripts might be long-non coding RNAs playing unknown regulatory roles, in keeping with the notion that almost two thirds of RNA molecules are non-coding transcripts within the cell [31], [32]. Although further work is needed to better understand why transcripts for the two NMNAT3 variants are present in HEK cells without being translated, our finding showing that they are also present in various human tissues at different levels underscores their potential functional relevance.

A widely appreciated dogma of cell biology is that mitochondria are impermeable to NAD [16], [33]. Several findings, however, indicate that this tenet should be revisited. First, both yeast and plant mitochondria have NAD transporters [34], [35]. Second, transporters for NAD precursors in the plasmamembrane or different cell organelles including mitochondria have not been identified so far. Third, inability of mitochondria to transport NAD has been only demonstrated in vitro, and it might be due to the fact that, under these experimental settings, transport systems are impaired or lack a co- or counter-molecule necessary for their functioning. Consistently, both yeast and plant mitochondrial NAD transporters work as nucleotide exchangers [34], [35]. Fourth, increase in the extracellular concentrations of NAD in cultured cells raises the dinucleotide content within their mitochondria and boosts cellular respiration [8]. Finally, prior work found evidence for metabolic state-dependent NAD fluxes through the inner mitochondrial membrane [36], [37]. These findings taken together, plus data of the present study indicating that exogenous NAD but not NMN, NR, NA or Nam prevents FKSG76-dependent mitochondrial NAD depletion, suggest that, akin to yeast and plants, yet-to-be defined mitochondrial NAD transporters are present in mammalian cells. Accordingly, we also found that increases of mitochondrial NAD pool by cytoplasmic NAD do not depend from matrix ATP contents. Data showing that mitochondrial NMNAT3 is absent in the organelle and cleaves NAD when artificially expressed further strengthen the hypothesis that the mitochondrial NAD pool is of cytoplasmic origin.

In the next future it will be worth investigating possible regulatory roles of non-coding FKSG76 and NMNAT3v1 mRNAs, as well as molecular identity of mammalian mitochondrial NAD transporters.

## Supporting Information

Figure S1 **Initial sequences of the ORF of FKSG76 and NMNAT3v1 plus a portion of their 5′UTR.** The initial ORF sequence of FKSG76 is shown in yellow (mitochondrial targeting sequence) and blue, whereas that of NMNAT3v1 is shown in blue. The final sequence of the 5′UTR of FKSG76 is shown in violet and red, whereas that of NMNAT3v1 is shown in violet and green. The ATG of the uORF is underlined at the beginning of the violet sequence.
(PDF)
