# Correction: Insight into Molecular and Functional Properties of NMNAT3 Reveals New Hints of NAD Homeostasis within Human Mitochondria

**DOI:** 10.1371/annotation/f5e6107f-a911-4c15-a881-7cb7e4946ff6

**Published:** 2013-12-11

**Authors:** Roberta Felici, Andrea Lapucci, Matteo Ramazzotti, Alberto Chiarugi

This article was republished on November 26th, 2013 due to data that was erroneously included in the Supporting Information. 

