# Supplementary material for: Insight into Molecular and Functional Properties of NMNAT3 Reveals New Hints of NAD Homeostasis within Human Mitochondria
Source: PLoS One. 2013 Oct 14;8(10):e76938. doi: 10.1371/journal.pone.0076938 (PMC3796565; doi:10.1371/journal.pone.0076938)
Supplement: Figure S1 — Initial sequences of the ORF of FKSG76 and NMNAT3v1 plus a portion of their 5′UTR. The initial ORF sequence of FKSG76 is shown in yellow (mitochondrial targeting sequence) and blue, whereas that of NMNAT3v1 is shown in blue. The final sequence of the 5′UTR of FKSG76 is shown in violet and red, whereas that of NMNAT3v1 is shown in violet and green. The ATG of the uORF is underlined at the beginning of the violet sequence. (PDF) [file pone.0076938.s001.pdf]

FKSG76

………TGCC<u>ATG</u>AAGCGGACTGCTGCTCCCTGGCTCCCACTCTGATCTGCTTTTCACTCTTGC
CTTGTCTCCCAATTAATAAGCAGGGTGTCCACTGGTACAAGGGTGGATGTGCCTGATAGATGA
AGAGCCGAGTACCTGTGGTGCTCCGGGCCTGTGGCTCCTGTAACCCCATCCCCAACATGCAC
CTGCGCATGTTTGAGGTGGCCAGAAATCACCTACACCAAACATGAATGTACCAGGT………

NMNAT3v1

………TGCC<u>ATG</u>AAGCGGACTGCTGCTCCCTGGCTCCCACTCTGATCTGCTTTTCACTCTTGC
CTTGTCTCCCAATTAATAAGGAATGTACCAGGT……….

Figure S1
